# Impact of Native and Nonnative Study Partners on Medical Students’ Confidence and Collaborative Strategies in Second Language Medical Dutch Learning

**DOI:** 10.1007/s40670-024-02138-1

**Published:** 2024-08-12

**Authors:** Hao Yu, S. Eleonore Köhler, Fatemeh Janesarvatan, Jeroen J. G. van Merriënboer, Maryam Asoodar

**Affiliations:** 1 School of Health Professions Education, Faculty of Health, Medicine & Life Sciences, Maastricht University, Universiteitssingel 60, 6229 ER Maastricht, The Netherlands; 2 Department of Anatomy and Embryology, Maastricht University, Maastricht, The Netherlands

**Keywords:** Confidence, Medical L2 learning, Network analysis, Simulated patient consultation

## Abstract

**Objective:**

This study explored how native and nonnative study partners impact medical students’ confidence, learning strategies, and perceptions of learning experiences in second language (L2) medical Dutch learning using Kolb’s experiential learning framework.

**Methods:**

Twelve third-year international bachelor medical students participated in a mixed-methods pre-post quasi-experimental design. Four students were paired with highly proficient native Dutch partners in a mixed group, and eight nonnative students formed pairs in a homogeneous group. The need satisfaction competence scale was used for pre- and post-tests, and individual interviews were analyzed for content and themes. Code co-occurrence and network analyses were carried out to identify the relationships between themes in the two groups.

**Results:**

Common themes in both groups included a positive atmosphere, collaboration, and confidence. The mixed group prioritized language learning and motivation, while the homogeneous group emphasized interpersonal relationships and feedback-seeking behaviors. Nonnative students in homogeneous groups gained confidence, while confidence of those in mixed groups decreased, possibly due to comparing themselves with native partners. Homogeneous groups have communication focused collaborative strategies, while the mixed group emphasized personal growth with fewer drawbacks.

**Conclusions:**

Our findings suggest that initiating the course with homogeneous nonnative student groups fosters collaboration and builds confidence among participants. However, to maintain motivation and further enhance language proficiency, it is advisable to introduce native Dutch partners at a later stage of the course. This approach allows students to benefit from both the positive collaborative atmosphere and interpersonal growth fostered in homogeneous groups, as well as the language learning and motivation gains associated with mixed-group experiences. Overall, our study highlights the importance of considering the stage of language learning and student needs in designing effective second language learning environments for medical students.

**Supplementary Information:**

The online version contains supplementary material available at 10.1007/s40670-024-02138-1.

## Introduction

Globalization leads to an increasing number of students studying and working abroad, necessitating proficiency in the local language for effective communication with colleagues and patients in the medical field. Medical interns face challenges in hospitals due to language barriers and ineffective communication. Fluency in Dutch is essential for international medical students in the Netherlands to effectively communicate with Dutch-speaking patients and colleagues in their future careers. Accordingly, courses teaching medical Dutch need to be introduced, and course designs needs to support efficient medical second language (L2) learning. Although medical simulations enhance students’ proficiency, having native speakers as learning partners offers experiential examples, feedback, and corrections for sentences and phrases. These approaches aim to equip students with the language skills necessary for successful medical practice in a Dutch-speaking environment [1, 2].

In medical L2 learning, study partners are often nonnative peers rather than native speakers of the language [3, 4]. In this study, the term nonnative indicates a person who is not a native speaker of the language in which the medical terms are studied. To assess the effects of nonnative or native study partners on language learning, we conducted a comparative study examining students’ levels of confidence in their language skills, their collaborative strategies, and learning experiences in homogeneous (study pairs of nonnative students) and mixed (study pairs comprising of a native and a nonnative student) groups. Varying proficiency levels between learning partners can result in differential impacts on students, and our study aimed to address this issue [3].

The fundamental question was which type of partner would optimally foster collaboration and language learning [5]. The medical Dutch course involved both native and nonnative Dutch speakers, to explore the potential impact of differences between nonnative and native speakers on language learning outcomes.

Pairing with a native speaker may enhance fluency and pronunciation, boosting students’ confidence in speaking like a native partner [6, 7]. Moreover, native partners can offer comprehensive language feedback, assisting nonnative students in identifying areas for improvement and boosting their confidence in L2 proficiency [8, 9]. Conversely, nonnative students may find it easier to relate to and receive empathetic support and encouragement from other nonnative students [10]. They can also share experiences and overcome language learning challenges at a similar pace, creating a positive learning experience with their partners [11–13].

**Fig. 1:**
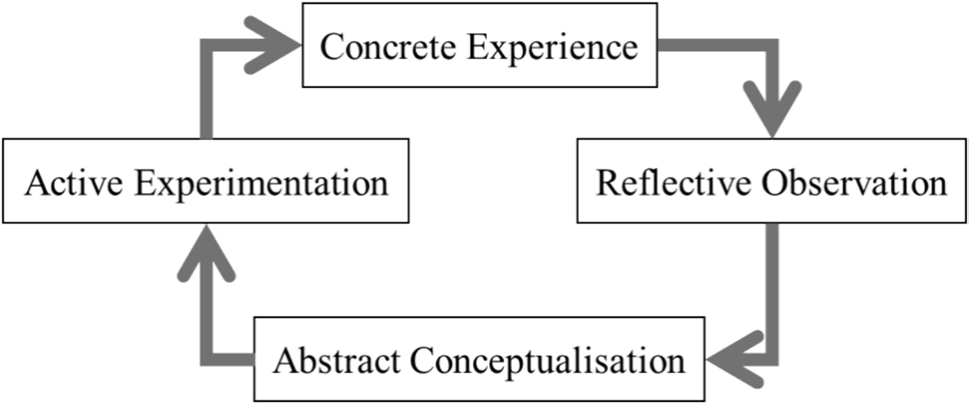
Model of Kolb’s (1984) experiential learning

Drawing from Kolb’s experiential learning theory [14], and considering relevant literature, we incorporated simulated consultations into the medical Dutch course to compare the effects of learning partners [1, 15, 16]. Kolb’s experiential learning theory proposes a learning cycle involving concrete experience, reflective observation, abstract conceptualization, and active experimentation [14] (see Fig. 1). Simulated patient consultations have proven effective for medical consultation and L2 learning, aligning with concrete experience and active experimentation. Pairing students for consultations fosters reflective observation and abstract conceptualization, offering valuable benefits through observational feedback and deep thinking [17–20]. Integrating these course design methods offers students’ opportunities to engage in real-life scenarios and reflective discussions with their partners. This fosters improved communication skills, language learning confidence, and collaborative benefits during consultations, enhancing professionalism in using medical Dutch [21–23].

Research questions were formulated based on the preceding discussion: (1) Does the confidence in medical Dutch significantly differ between students learning in mixed and homogeneous groups? It was hypothesized that students paired with native Dutch-speaking partners would display higher confidence than those paired with nonnative partners. (2) Are there variations in perceived collaborative strategies and learning experiences between mixed and homogeneous groups? The hypothesis suggested that the mixed group, where nonnative students were paired with native partners, would be more open to feedback and exhibit a feedback-centered learning experience, while the homogeneous group might establish stronger partner relationships with closer bonds or partnership between nonnative partners.

## Method

### Design and Participants

**Fig. 2:**
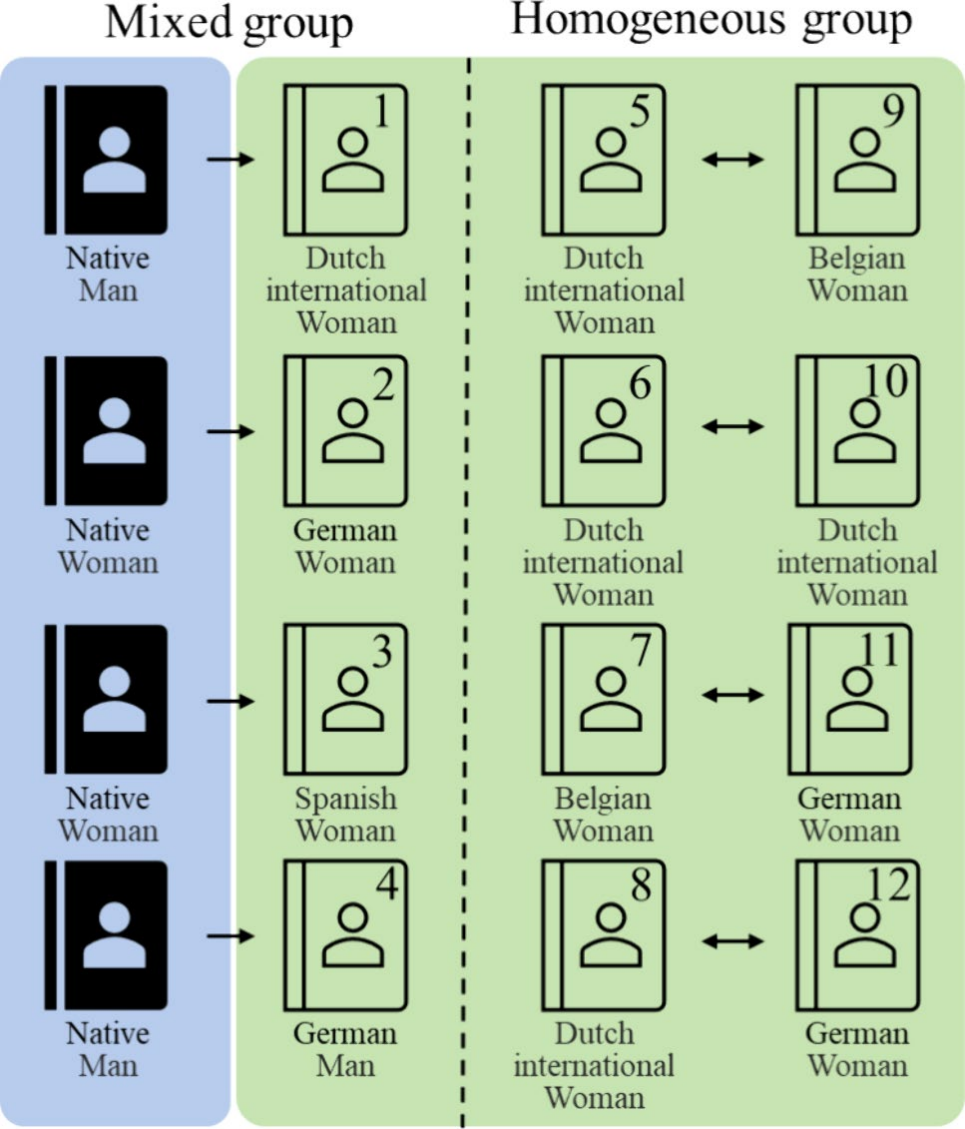
Background information on the quasi-experimental groups. Native facilitators are indicated in blue, and nonnative participants in green. Gender, student number, and nationality are indicated in the image. Mean age in both groups was similar (22 years)

This mixed-methods study utilized a pre- and post-test comparative design with 12 third-year international medical students (mean age = 22.3 years, range from 20 to 28 years) enrolled in a medical Dutch course at Maastricht University (Fig. 2). Four exceptionally proficient native Dutch-speaking students, exempted from the course based on a rigorous language evaluation, were invited back as native partners to assist the medical Dutch learning of the other students. The mixed group consisted of these four native partners randomly paired with four of the 12 students, while the remaining eight students formed four homogeneous groups. All students attained a proficiency level of at least B2 in Dutch, according to the Common European Framework of Reference for Languages (CEFR), which signifies their ability to engage in intricate social dialogues, comprehend and analyze sophisticated Dutch texts and books, and articulate complex thoughts and viewpoints in writing. Level B2 is the standard for foreign diploma holders who aspire to pursue higher professional education in the Netherlands. Additionally, the course was conducted in Dutch, and they possessed a minimum of 2 years of practical experience in simulated patient consultation sessions conducted in English, which allowed them to focus on Dutch language learning during the course. The nonnative participants were German (4), French (2), Spanish (2), Finish (1), Irish (1), Romanian (1), and Italian (10) students.

Participants were compensated with 20 euros per hour for their additional time spent cooperating with the researcher, which included completing questionnaires and participating in interviews after the course.

#### Course Structure and Pairing of Students

**Fig. 3:**
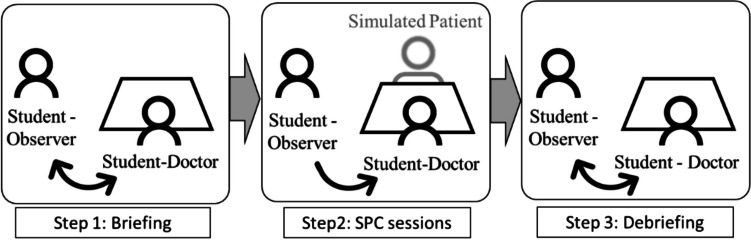
Simulated patient consultation component

The 15-week program comprised 2-h Dutch medical teaching sessions each week, covering advanced medical terminology, grammar, consultation structures, and example videos. Additionally, there were three 30-min simulated patient consultation (SPC) sessions in the 8th, 11th, and 14th weeks. Each student had a designated study partner for these sessions, as shown in Fig. 3.

During the SPC sessions, each member of a study pair could choose to be either the student-doctor or student-observer (Fig. 3, step 2). After the session, student-doctors received feedback from their student-observers in a debriefing session (Fig. 3, step 3). Roles were then switched, and steps 2 and 3 were repeated. In total, each student acted as a student-doctor and student-observer three times during the SPCs.

### Materials

#### Need Satisfaction Competence Scale in Pre- and Post-tests

The need satisfaction competence scale (NSCS) was used in both pre- and post-tests to assess students’ confidence in learning medical Dutch as their L2. Each item was rated on a 7-point Likert scale, with responses ranging from 1 (strongly disagree) to 7 (strongly agree), i.e., “In my studies, I feel highly effective at what I do.” (available as Supplementary Digital Appendix 1). The Cronbach’s *α* reliability coefficient for the NSCS was 0.81.

#### Perceived Collaborative Strategies

To collect information about collaborative strategies, students were individually interviewed. The interviews were aimed at understanding their opinions about their partner’s proficiency in medical Dutch. Participants were asked questions like “How would you describe your partner’s ability to use medical Dutch?” Furthermore, students were asked to share the specific methods their partners used to support their language learning. Questions like “What strategies do you and your partner use to help each other?” were posed.

#### Individual Semi-structured Interviews

To reduce the likelihood of social desirability bias [24], participants were invited individually to the semi-structured interviews. Each interview guide included questions related to the four components of the study:

1. Learning atmosphere (e.g., “Can you describe the atmosphere when your partner was with you during the course?”),
2. Partner support (e.g. “How did you feel about the progress of your consultation ability in relation to your partner?”),
3. Confidence (e.g., “Can you recall when you felt confident in medical Dutch and which part of the medical Dutch course contributed to this feeling?”),
4. Language learning (e.g., “How did your partner help you with learning medical Dutch?”). (Ava for the full interview guide)

### Procedure

A pre-test scale was used to assess the initial level of confidence among students after the first week of the course. At the end of the course, students completed the post-test questionnaire and participated in the interview session the following week.

### Data Analysis

The individual semi-structured interviews underwent content and thematic analyses to uncover the collaborative strategies and learning experiences underlying both the mixed and homogenous groups [25, 26]. The collaborative strategies were independently identified by two researchers from the field of education (H.Y. and M.A.) and were summarized into six categories, available as supplemental digital appendix 4. Two researchers (H.Y. and F.J.) independently coded the interview data, compared and discussed the resulting codes and themes, and resolved any discrepancies in two team meetings. The thematic findings underwent a code co-occurrence analysis, which allowed the generation of nodes and edges for subsequent network analysis [27–29]. The network analysis was based on data from the code co-occurrence matrix and coefficients of codes and was available as supplemental digital appendix 3. A force-directed graph was provided to visualize the structure of the networks.

## Results

Research question 1 was aimed at comparing the difference in confidence levels between participants in the mixed and homogeneous groups. The findings from the pre- and post-test NSCS questionnaires provided evidence to support evidence addressing this research question.

### Students’ Confidence in Learning Medical Dutch

We analyzed the NSCS results to determine the confidence of the participants in their medical Dutch at the beginning and the end of the study (Table 1). The Mann–Whitney *U* test result and the normalized gain score revealed that the confidence of the mixed group decreased marginally (*U* = 3.85, *Z* =  − 0.55, *p* = 0.58, *r* = 0.28) resulting in a small negative normalized gain (N-gain =  − 10.7%, SD = 0.25; Table 1) while the confidence of the homogenous group improved substantially (*U* = 15.50, *Z* =  − 2.21, *p* = 0.03, *r* = 0.38) and showed a positive normalized gain (N-gain = 11.3%, SD = 0.08).

**Table 1:** Confidence of nonnative students in the mixed and homogenous groups

Confidence	Mixed (*n* = 4)		Homogenous (*n* = 8)	
	Median	IQR	Median	IQR
Pre-test	5.53	1.33	4.67	1.41
Post-test	5.33	2.67	5.83	1.17
N-gain	− 10.7%	± 0.25 (SD)	11.3%	± 0.08 (SD)

### Perceived Collaborative Strategies

Research question 2 first investigated collaborative strategies in mixed and homogeneous groups using content analysis. Responses to the interview question “How did your partner affect your medical Dutch learning?” yielded a total of 238 responses on strategies. Among these, mixed groups reported 58 times on strategies (24% of 238), while homogeneous groups reported 180 times on strategies (76% of 238), indicating higher strategy exchange in the homogeneous groups. Available as supplemental digital appendix 4, collaborative strategies:

1. Language use in SPC, covering grammar and word usage during sessions.
2. Communication skills, including tone, facial expressions, empathy, questioning style, and other communication-related aspects.
3. Partner comparison, involving feedback or observations comparing one’s performance with their partner.
4. Interaction and feedback, concerning the nature of feedback received, including positivity, constructiveness, warnings or criticisms, and knowledge sharing.
5. Personal growth, referring to flexible thinking, enhanced learning effectiveness, and self-recognition of personal growth.
6. Drawbacks, encompassing challenges like language communication difficulties, anxiety, lack of feedback, and ineffective feedback.

**Fig. 4:**
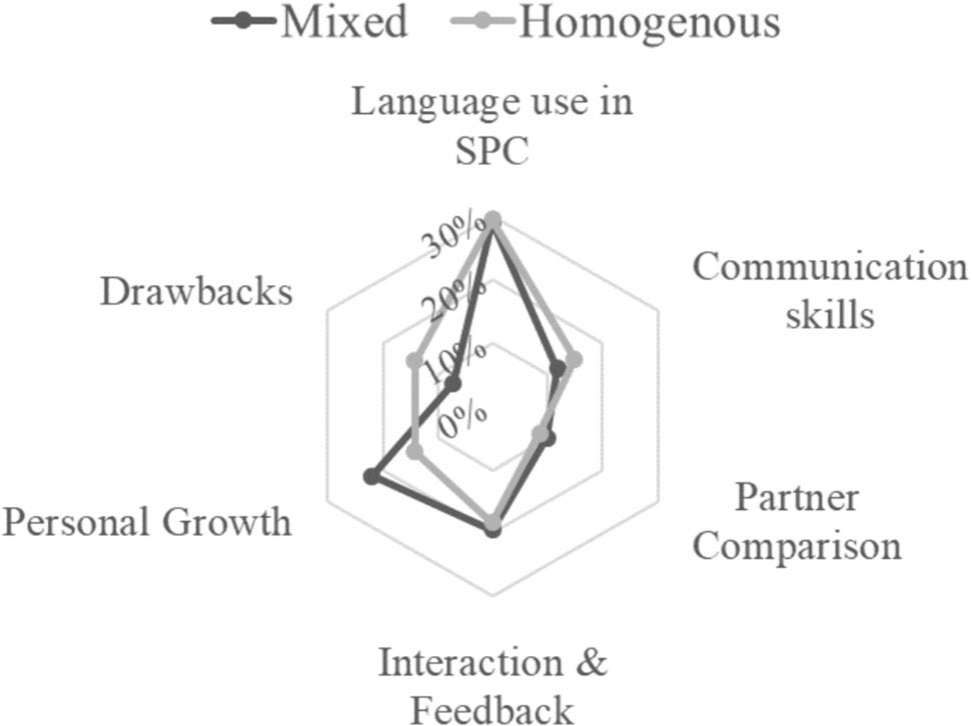
Radar plot of collaborative strategies

The radar plot in Fig. 4 visually represents the six dimensions. Both groups shared similar collaborative strategies in language use in SPC, partner comparison, and interaction and feedback. The homogenous group showed a slightly higher prevalence in communication skills, while the mixed group emphasized personal growth more. Additionally, the homogenous group exhibited a higher occurrence of drawbacks (available as Supplemental Digital Appendix 3).

### Perceived Learning Experience

**Fig. 5:**
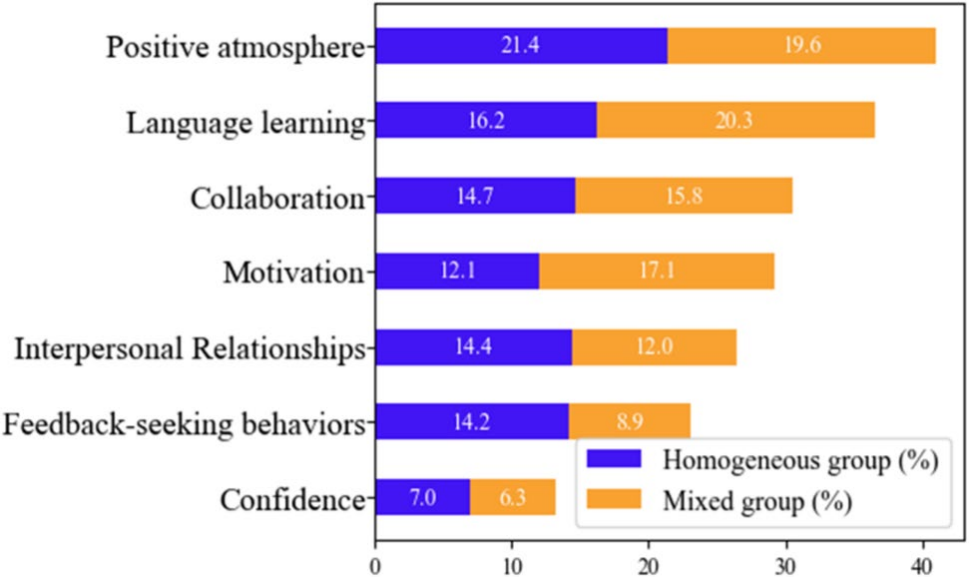
Distribution of themes in the homogenous and mixed groups in %

Thematic analysis was conducted to answer the second part of research question 2, namely to understand students’ learning experiences and identify potential connections between themes in the network analysis. Seven themes emerged from both mixed and homogenous groups, presented as percentages in Fig. 5. Some themes were addressed by both groups to a similar extent, while other themes were more dominant in one group.

**Fig. 6:**
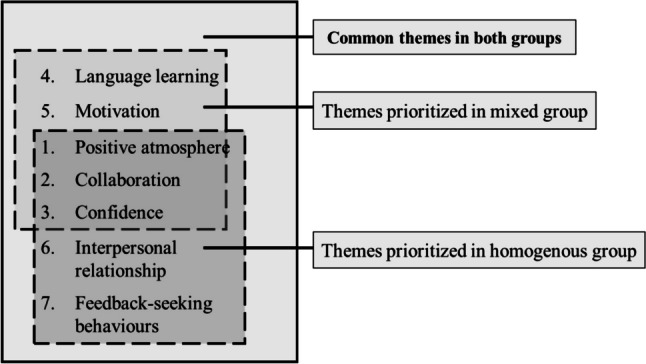
Common and prioritized themes

The distribution of these themes is visualized in a diagram (Fig. 6). The three themes positive atmosphere, collaboration, and confidence showed comparable percentage of codes across both groups. In each group, two separate themes were prioritized. The homogenous group reported a higher frequency of themes related to interpersonal relationships and feedback-seekin*g* behaviors while the mixed group demonstrated a higher frequency of themes associated with language learning and motivation.

#### Common Themes of Both Groups

##### Positive Atmosphere

The positive atmosphere emerged as one of the most frequently mentioned themes with 38 occurrences, with highest frequencies for codes associated with positive feedback. Additionally, codes such as positive reinforcement, appreciation, and codes related to attitude were highly valued within this theme (available as Supplemental Digital Appendix 2, Table S1). Student 3 and student 6 stated the following:

So it’s not someone that makes you just feel all the time stressed or something like that, but like encouraging even if you are not doing so well or whatever, because obviously everyone has their own process. (student 3 from mixed group)

I would say that we have a good atmosphere among us, as we’re also friends outside of university. It feels friendly and respectful, and we’re always trying to help each other. We don’t make fun of each other, but we do point out areas for improvement while also acknowledging the things that are going well. That way, we stay positive and don’t get stuck on the negatives (student 6 from homogenous group).

##### Collaboration

The theme of collaboration encompassed the dynamic process of guiding individuals towards shared objectives, fostering cooperation and problem-solving, and promoting synergy within the group. The thematic analysis revealed codes that aligned with the collaborative strategies shown in Fig. 4. These codes included interaction and feedback, which were mentioned 23 times. Peer support in language learning and communication was mentioned 19 times, while observations were mentioned 12 times. Other supporting codes can be found in supplemental digital appendix 2, Table S1. Student 2 and student 12 expressed the following:

Sometimes, my peer could see certain aspects about how my talk was, or if I misuse a word or something, it can be done subconsciously so in those aspects, the observing student helped with my Dutch language. (student 2 from mixed group)

I think it’s really useful to track one person’s impact on the improvement through pairing up. Because right now we’re not a group of different people every time. So we can keep track and it’s a lot easier to see where we started. (student 12 from homogenous group)

##### Confidence

The theme of confidence emerged as a significant aspect in this study, with 27 codes identified in the homogenous group and 10 codes in the mixed group. The themes identified emphasized the positive impact of peer collaboration on boosting students’ confidence and highlighted the importance of confidence in the learning process. Specifically, confidence gained from peer collaboration was mentioned 14 times, while confidence derived from students’ improvement and success was mentioned 18 times. Furthermore, additional codes can be found in supplemental digital appendix 2, Table S1. As quoted by students 11 and 2:

It is nice to have someone who knows me and knows my level of Dutch. Because we always know that they are just there to help you, they are not like to judge you or grade you or anything, and so, yeah, it increases my confidence to have someone that I know next to me. (student 11 from homogenous group)

So now her feedback to me was always ‘you are doing good’ For that she would tell me what words I could change, how to do it better, and that was really nice and then she was always very sweet, and she made notes during my SPC, … that was helping a lot and gave me confidence. (student 2 from mixed group)

#### Prioritized Themes of Mixed Group

In addition to the common themes identified in both the mixed and the homogenous group (Fig. 6), the themes of language learning and motivation were highly prioritized in the mixed group, with a higher percentage of codes on that topic reported compared to the homogenous group.

##### Language Learning

This theme described the effect of students supporting each other, providing feedback, and offering necessary assistance in learning Dutch. The collaborative strategies of language use in SPC and personal growth dominantly described the theme of language learning. A slight difference was found between the groups. Specifically, the code of language barriers (mentioned 7 times) highlighted the challenges that emerged from students comparing their language proficiency with native partners. It was observed that in mixed groups, the quality of feedback provided was more in-depth and constructive compared to the homogeneous group.

In general, in mixed groups, three codes, namely Dutch learning (mentioned 11 times), language barriers (mentioned 7 times), and language improvement (mentioned 6 times), predominantly contributed to this theme (available as Supplemental Digital Appendix 2, Table S2). An example quotation from student 3 illustrated this.

My partner always gives me feedback when I need it. It is better to say that when it is not comfortable talks in that practice, they can then try to give feedback more on the structure of the consultation. You obviously have to understand that the different backgrounds also influence. So you try to make the best of it, obviously and still get helps from your partner. (student 3)

On the other hand, in homogenous groups, the focus primarily revolved around Dutch learning (mentioned 42 times) and language improvement (mentioned 10 times). Here is an example quotation from student 11.

Maybe student 7 and I are more on the same level because we are both German. It’s very similar to Dutch. So it’s also like we also learn it in the same in the same way. And we have the same struggles with like words that are not that similar or grammar that’s different. (Student 11)

##### Motivation

This theme described how students were motivated, either by external factors or through self-motivation, during the course. In the mixed group setting, the theme of motivation was predominantly supported by the presence of a native partner (mentioned 16 times, available as Supplemental Digital Appendix 2, Table S2). A quotation from student 3 illustrated this aspect.

I think even I was a bit insecure in the beginning of my medical Dutch course, and she was giving me good feedback, ‘you did fine’, ‘don’t worry, and it’s good’ and that put it into perspective and I felt a lot better afterwards. Because she know that I trust her, and all that ‘she’s good’ or when she says, ‘you are doing fine’, I believe her. (student 3)

On the other hand, in the homogenous group, the primary code that stood out was “motivational SPC setting” (mentioned in the quotation from student 5).

I think the patient is the main person and the most important person in that situation. I think that like the patient being that really motivates me to do a good job. And I want to make a practice for being a good doctor later. So I don’t really think about my peers being there. I just like think about the patient was there and try to do my best in and pretending I’m a doctor and they are patient. So I think that’s like my main motivation and also what I focus on. (student 5)

#### Prioritized Themes of Homogenous Group

In the homogenous group, the themes of interpersonal relationships and feedback-seeking behaviors had a higher reporting rate in interviews compared to the mixed group (Fig. 6). These themes were also found in collaborative strategies; for example, partner comparison and interactive and feedback strategies were also coded in the themes of interpersonal relationships and feedback-seeking behavior.

##### Interpersonal Relationships

The theme of interpersonal relationships encompassed a diverse range of codes that displayed variations across different groups. Within the homogenous groups, the code of social interaction and support was mentioned 14 times. Additionally, communication skills (7 times) and friendship (7 times) emerged as important components (available as Supplemental Digital Appendix 2, Table S2). Two example quotations from students 6 and 7:

It is nice when you have someone accompany you in the room other than just a patient. So you can always ask for time out, or at the end, they will know a bit more about our courses, like the structure and stuff that the patient doesn’t necessarily know. ... I think she has a good standard of speaking Dutch, and she can also understand most things. So I think her Dutch is the same level as mine around. (student 6)

We spend time doing the SPC together; we become closer over the time…. I think it is a bonding thing if you study something together, and you learn together, and then you kind of become close. So it improves our relationship and the overall atmosphere. (student 7)

In contrast, in mixed groups, one prominent factor observed was comparison, 5 times. Additionally, other codes such as comradery, sense of belonging, social norms, and pressure were mentioned 2 times across all the interviews, as highlighted in the quotation from student 4.

Sometimes it can be a bit unfair when you’re all in the same class, but then someone is already on a higher level, then, we are also considering this have a beneficial effect because they already speaking good, they can constantly giving more peer feedback. (student 4)

##### Feedback-Seeking Behavior

The theme of feedback-seeking behaviors highlights the specific codes that are unique to different groups. In the context of homogenous groups, the importance of seeking feedback by drawing upon experiences from their collaboration was mentioned 27 times, which also overlays with collaborative strategies of interaction and feedback, as illustrated in the example quotation from student 10. Further details regarding the rest of the codes can be found in supplemental digital appendix 2, Table S3.

I think, in fact, I affected their learning in the same way, because we are just in a group together and we do exercises also together and then. We can also learn from each other’s useful tips. They can also inspire that when I see my partner has a really nice way of speaking or uses a specific phrases. Then I can also pick up on that and also incorporated into my consultation. Maybe I think that’s really nice. Also, that feedback is really useful, because sometimes I’m also not aware of certain things during the consultation. (student 10)

Differently, in mixed groups, the prominent codes related to feedback were feedback constructiveness (5 times) and experience-based feedback (4 times), as indicated in the quotation from student 4.

I think if it appears to be giving feedback the way natives did, yeah, quite constructive because they’re not just saying you are good. They could really give me an example of what is not going right. They can really pinpoint it and give me the reason for it. (student 4).

### Network Analysis of Themes

**Fig. 7:**
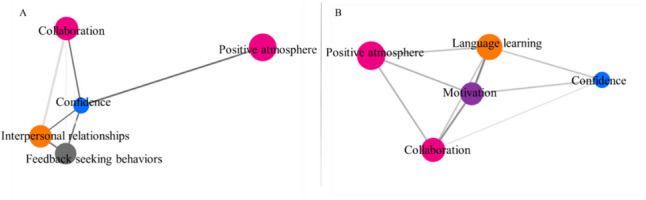
Force-directed network of themes for the homogenous group (**A**) and mixed group (**B**)

The thematic analysis findings led to the exclusion of language learning and motivation themes from the homogenous group, and interpersonal relationship and feedback-seeking behavior themes from the mixed group in the network analysis. These themes were considered to have less meaningful implications for each group. The code co-occurrence matrix and coefficients were then utilized to create the nodes and edges of the force-directed graph, illustrating the codes (available as Supplemental Digital Appendix 3, Fig. S1, Fig. 7A).

The network properties of nodes and edges were presented using code concurrence and code coefficients (Table 2). The weighted in-degree (wd) indicates a node’s connectivity and importance within the network, while the PageRank (PR) value measures network centrality, indicating the significance of a specific node.

**Table 2:** Network metrics of the homogenous and mixed groups

Network metrics	Homogenous group	Mixed group
Nodes (*N*)	5	5
Edges (*N*)	10	9
In degree (*M* ± SE)	4.0 ± 0	3.6 ± .49
Weighted in-degree	.24 ± .05	.12 ± .03
PageRank (*M* ± SE)	.14 ± .02	.14 ± .02
Diameter	1	2
Density	1	.90
Modularity	0	0
Communities (*N*)	1	1
Path length (*M*)	1	1.1

Consistent PR values were observed for the “themes” at both mixed group and homogenous group levels, indicating similar centrality and importance throughout the networks. Additionally, all networks showed a single prominent community with comparable density and similar diameter, and the average path length indicated similar interconnectedness within the networks.

In the homogenous group, despite the low code amount, the node “confidence” (wd = 0.14, PR = 0.106) played a central role in connecting to other nodes in the five-theme network community. Two distant nodes, “positive atmosphere” (wd = 0.28, PR = 0.146) and “collaboration” (wd = 0.27, PR = 0.158), were linked to “confidence.” The nodes “interpersonal relationship” (wd = 0.25, PR = 0.142) and “feedback-seeking behaviors” (wd = 0.26, PR = 0.149) were also closely connected to confidence.

In the mixed group, a single five-theme network community was identified, with the central node being “motivation” (wd = 0.17, PR = 0.142). The nodes “positive atmosphere” (wd = 0.08, PR = 0.145) and “confidence” (wd = 0.09, PR = 0.106) played more peripheral roles in the network compared to the nodes “language learning” (wd = 0.12, PR = 0.149) and “collaboration” (wd = 0.12, PR = 0.159).

## Discussion

For research question 1, our findings indicate that the presence of supportive and encouraging partners boosts students’ confidence, playing a significant role in contributing to a notable improvement in their performance [30]. Students in mixed groups initially exhibited high self-confidence during the pre-test but showed a decrease in confidence after the course, reflected in a negative normalized gain score. This drop in confidence was likely due to their reliance on positive feedback from native partners and the collaborative strategy of comparing themselves to their partners. In contrast, the homogeneous group likely did not experience such a drop in confidence as they were not directly comparing themselves to students with high performance levels. It is worth noting that there was an initially high confidence level in the nonnative students in the mixed group, which we infer was influenced by the initial encounters and assistance from native partners. The native partners consistently outperformed the nonnative students throughout the learning process, leading to a basis for comparison or appreciation among the students [31, 32]. The stark contrast in performance could have led to feelings of inadequacy and self-doubt, ultimately undermining their self-confidence [33]. Constantly being overshadowed by their native partners could have eroded the students’ belief in their own abilities, resulting in a decrease in confidence over time [33, 34].

The similar proficiency levels in medical Dutch within the homogenous group likely facilitated their use of collaborative strategies by enabling better mutual understanding and support. Our study identified six collaborative strategy domains. Both groups emphasized language use, progress monitoring, and feedback. However, the homogenous group focused more on communication skills, likely due to their shared linguistic proficiency and understanding of challenges, enhancing intra-group communication. Moreover, the mixed group focused more on personal growth as collaborative strategies, which included self-improvement, reflection, analytical thinking, and confidence building (available as Supplemental Digital Appendix 4). We posit that this can be attributed to the role modeling effect, as documented in previous studies [35–37]. In the mixed group, native peers serve as valuable learning models, inspiring students to reflect on their own skills and qualities to emulate the successful behaviors and characteristics exhibited by these role models [37]. Inspired by their native partners, students in the mixed group aimed to reach the level of competence demonstrated by these role models. Consequently, the homogenous group faced more challenging drawbacks than the mixed group [38, 39].

To understand the theme construct, we used network analysis to model relationships in both the homogenous and mixed groups in line with previous research that thoroughly investigated these themes to validate their construct [38, 40–44]. In the homogenous group, confidence was central and closely linked to other themes, while motivation was central in the mixed group (Fig. 7). These findings align with Kolb’s theory, which emphasizes the importance of a motivational environment and building students’ learning confidence to enhance their learning experiences [45, 46]. The use of native or nonnative partners in the course, as guided by Kolb’s theory and thematic analysis outcomes, effectively accounts for the differences in network themes observed between the mixed and homogeneous groups.

### Implementation

Our findings align with previous findings that nonnative partners contribute to creating a collaborative and positive atmosphere, enhancing concrete experience in a supportive learning environment, and boosting confidence in learning [47, 48]. Students’ confidence was strengthened through a combination of positive feedback from their partners’ observations and self-reflection on their progress and development during language learning [49].

Based on Kolb’s theory, we delved deeper into how these themes correlate with the students’ perceived learning experiences [48, 50, 51]. Themes like interpersonal relationships and feedback-seeking behavior align with the reflective observation stage [47, 52, 53]. During this stage, students engage in interactions, seek feedback, and learn from interpersonal dynamics, fostering valuable experiences for reflection and growth [54]. The mixed group excelled in abstract conceptualization and active experimentation. Language learning and motivation were linked to abstract conceptualization, where students develop knowledge and strategies while experiencing increased motivation [13]. Active experimentation, focusing on language practice and immersion, positively influences students’ motivation during language learning [52, 55].

**Fig. 8:**
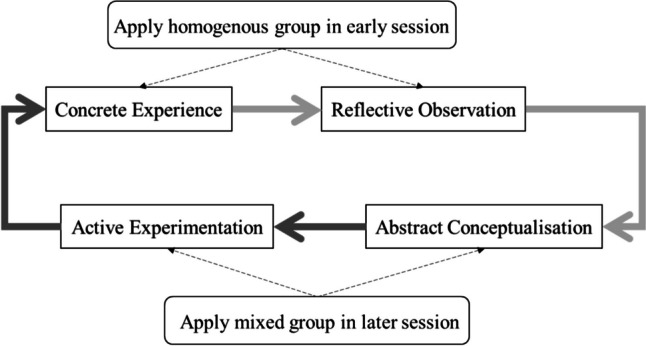
From prioritized theme to prioritized stages

We recommend using nonnative partners in the course’s initial stage to foster familiarity, collaboration, and confidence in overcoming shared challenges. In practice, native partners should be introduced in the later stages of the course to facilitate learning with highly competent and motivated partners, leading to improved language skills, overcoming drawbacks, and advancing to higher levels of medical Dutch competence (Fig. 8). In future research, we suggest considering both native and nonnative study partners to improve curriculum design, given the limited native partner resources. This could help boost communication and performance in specialized fields like education, law, technology, and business.

### Limitations and Future Work

Our study had limitations that may have influenced the interpretation of results and discussions. The small sample size, particularly in the mixed group (4 pairs), led to higher standard deviation in the normalized gain score for research question one, making it difficult to definitively establish a decrease in the gain score. Future studies could replicate our design with a larger sample size to strengthen and expand our conclusions. The gender distribution in both groups was imbalanced, with a higher number of female students (*n* = 13) participating compared to male students (*n* = 3). In the international track of medicine program, more women (80%) enlisted than men. In our study, there were more women than men in both groups, especially in the homogeneous group. It is unclear whether gender could play a role in the learning outcomes. We hope future research will explore similar contexts with a balanced gender composition. The students and native-speaking partners were all familiar with each other since they were enrolled in the same program with 50 students/year at the university and have studied simulated patient consultation in English for 2 years. For other designs, students could become familiar with each other in small groups before pairing them up. Additionally, the unique nature of each network in the network analysis, along with differing parameters used to describe network attributes across groups, made direct comparisons challenging. Lastly, there was a discernible tendency to emphasize the advantages of native partners, although our study primarily did not involve interviewing native partners. We recommend that future study also conduct interviews or surveys on the native partners’ perceptions for gaining further understanding of near-peer teaching and mentoring.

## Conclusion

This study found significant differences in perceived learning experiences between mixed and homogeneous groups. The homogeneous group showed increased self-confidence, while the mixed group experienced a slight decrease due to comparisons with native peers but showed more motivation and experimentation. Accordingly, nonnative partners are recommended for the initial stage of the course to boost students’ self-confidence, while native partners can aid rapid language improvement and motivation in the second half. In future research, we recommend exploring the optimal use of native and nonnative study partners in curriculum design, considering their unique advantages and limitations.

## Supplementary Information

Below is the link to the electronic supplementary material.

Supplementary file1 (DOCX 18 KB)

Supplementary file2 (DOCX 18 KB)

Supplementary file3 (DOCX 621 KB)

Supplementary file4 (DOCX 31 KB)

## Data Availability

The datasets generated and/or analyzed during the current study are available in the Dataverse repository (https://dataverse.nl/).

## Abbreviations

L2: Second language
CEFR: Common European Framework of Reference for Languages
NSCS: Need Satisfaction Competence Scale
