# Supplementary material for: Impact of Native and Nonnative Study Partners on Medical Students’ Confidence and Collaborative Strategies in Second Language Medical Dutch Learning
Source: Med Sci Educ. 2024 Aug 12;34(6):1445–55. doi: 10.1007/s40670-024-02138-1 (PMC11699018; doi:10.1007/s40670-024-02138-1)
Supplement: Supplementary file 1 — Supplementary file1 (DOCX 18 KB) [file 40670_2024_2138_MOESM1_ESM.docx]

**Supplemental Digital Appendix 1**

**Article Title:** Impact of native and nonnative study partners on medical students’ confidence and collaborative strategies in second language medical Dutch learning

**Journal Name:** Medical Science Educator

**Author Names:** Hao Yu^1*^, S. Eleonore Köhler^2^, Fatemeh Janesarvatan^1^, Jeroen J. G. van Merriënboer^1^, Maryam Asoodar^1^

**Affiliation:** ^1^School of Health Professions Education, Faculty of Health, Medicine & Life sciences, Maastricht University, the Netherlands

^2^Department of Anatomy and Embryology, Maastricht University, Maastricht, The Netherlands

E-mail address of the corresponding author: [h.yu@maastricht](mailto:h.yu@maastricht)university.nl

**Instruments**

***1.Need satisfaction competence scale (7-point Likert scale)***

1. I feel I am very good at the things I do

2. I feel highly effective at what I do

3. I feel I can accomplish even the most difficult tasks

1. ***Individual Semi-Structured Interviews***

**Demographic**

1. Who is your pair?

2. Can you describe your impression on your pair’s medical Dutch language ability?

3. Can you describe your impression on your pair’s consultation ability?

**Pair strategy**

1. Can you describe you and your pair’s strategies on helping each other during the simulated consultation? (How do you collaborate with your pair? Please describe how you and your pair help each other. Why did you choose this strategy and why do you think it helps?)

**Supportive atmosphere**

1. Can you describe the atmosphere when you and your pair give feedback to each other? (Ask what, and why for more information).

**Consultation model**

1. Having a pair involved in your consultation is not as realistic as your career consultation environment. What part of the “consultation model” have been influenced by having a peer involved in your consultation? (Ask both good and bad influences)

**Progress**

1. How do you evaluate the changes in your achievement caused by having a pair involved? (Why do you say so?)

**Medical Dutch learning**

1. What are the advantage and disadvantage when your pair involved in your medical Dutch language learning? (Please be specific in your explanation.)

**Intrinsic motivation**

9. How does the pair involved in your medical consultation influence your intrinsic motivation? (What is the reason?)

a) Does pair involvement influence your intrinsic motivation to know new knowledge? (What is the reason?)

b) Does pair involvement influence your intrinsic motivation in engaging in learning activities? (What is the reason?)

c) Does pair involvement influence your intrinsic motivation for pursuing a flow, excitement, or pleasure for learning? (What is the reason?)

d) Do you consider that the pair-setting demotivating your learning? (What is the reason?)

**Need satisfaction**

10. Does pair involvement influence your voluntariness? Do you feel you autonomously want to learn consultation or medical Dutch-related knowledge? (Please explain the reason for your answers.)

11. Does pair involvement influence your confidence in learning medical Dutch or consultation? (Please explain the reason for your answers.)

12. Does pair involvement influence your relationship with your pair/peer/teacher/the simulated patient? (Please explain the reason for your answers.)
