# Supplementary material for: Impact of Native and Nonnative Study Partners on Medical Students’ Confidence and Collaborative Strategies in Second Language Medical Dutch Learning
Source: Med Sci Educ. 2024 Aug 12;34(6):1445–55. doi: 10.1007/s40670-024-02138-1 (PMC11699018; doi:10.1007/s40670-024-02138-1)
Supplement: Supplementary file 2 — Supplementary file2 (DOCX 18 KB) [file 40670_2024_2138_MOESM2_ESM.docx]

**Supplemental Digital Appendix 2**

**Article Title:** Impact of native and nonnative study partners on medical students’ confidence and collaborative strategies in second language medical Dutch learning

**Journal Name:** Medical Science Educator

**Author Names:** Hao Yu^1*^, S. Eleonore Köhler^2^, Fatemeh Janesarvatan^1^, Jeroen J. G. van Merriënboer^1^, Maryam Asoodar^1^

**Affiliation:** ^1^School of Health Professions Education, Faculty of Health, Medicine & Life sciences, Maastricht University, the Netherlands

^2^Department of Anatomy and Embryology, Maastricht University, Maastricht, The Netherlands

E-mail address of the corresponding author: [h.yu@maastrichtuniversity.nl](mailto:h.yu@maastrichtuniversity.nl)

**Additional results**

**TABLE: *S1 Themes Addressed Equally by Both Groups through Code Composition***

| Themes | codes |
| --- | --- |
| Positive atmosphere | positive feedback (38), positive reinforcement (8), supportiveness (7), appreciation (7), gratitude (5), positive attitude (5), respect (5), positivity (4), trust (4), strengths (3), helpfulness (4), safe environment (3), consistency (3), accountability (2), honestly (2), inclusivity (2), neutral attitude (2), ... |
| Collaboration | interaction and feedback (23), peer supports (19), observation (12), communication (7), learning from others (3), approval (2), validation (2), share experience (1), feeling supported (1), acknowledging others, ... |
| Confidence | Confidence in learning (14), improvement (12), self-confidence (7), success and building confidence (6). |

Note: Code occurrences are shown in parentheses, code occurrences = 1 are omitted, but indicated by "...".

**TABLE: S2 *Prioritized*** ***themes of Mixed Group***

| Themes | Codes from mixed groups |
| --- | --- |
| Language learning | language barriers (7), cultural difference (3), language difficulties (3), Dutch learning (11), language improvement (6), preparation (2). |
| Motivation | motivating partner (16), self-motivated (3), self-doubts (4), performance anxiety (2) |

Notes: Code occurrences are shown in parentheses, code occurrences = 1 are omitted by marking "...".

| Themes | Codes from homogenous groups |
| --- | --- |
| Language learning | Dutch learning (42), language improvement (10), cultural influence (4), language barriers (2),  acquisition (2), preparation (2), … |
| Motivation | motivational SPC setting (25), self-doubts (4), encouragement (5), autonomy (2) |

**TABLE: *S3 Prioritized themes of Homogenous Group***

| Themes | Codes from homogenous groups |
| --- | --- |
| interpersonal relationships | Communication skills (7), social support (7), social interaction (7), friendship (7), comparison (5), dynamics (3), bonding (2), comradery (2), community (2), … |
| feedback-seeking behaviors | Experience based feedback (27), constructiveness (6), autonomy and desire of feedback (4) |

Note: Code occurrences are shown in parentheses, code occurrences = 1 are omitted by marking "...".

| Themes | Codes from mixed groups |
| --- | --- |
| interpersonal relationships | Comparison (5), comradery (2), relationship (2), sense of belonging (2), social norm and pressure (2), bonding (1), respectful communication (1), … |
| feedback-seeking behaviors | Constructiveness (5), experience-based feedback (4), appreciation and helpfulness of feedback (2) |

Note: Code occurrences are shown in parentheses, code occurrences = 1 are omitted by marking "..."
