# Supplementary material for: Impact of Native and Nonnative Study Partners on Medical Students’ Confidence and Collaborative Strategies in Second Language Medical Dutch Learning
Source: Med Sci Educ. 2024 Aug 12;34(6):1445–55. doi: 10.1007/s40670-024-02138-1 (PMC11699018; doi:10.1007/s40670-024-02138-1)

**Supplemental Digital Appendix 3**

**Article Title:** Impact of native and nonnative study partners on medical students’ confidence and collaborative strategies in second language medical Dutch learning

**Journal Name:** Medical Science Educator

**Author Names:** Hao Yu^1*^, S. Eleonore Köhler^2^, Fatemeh Janesarvatan^1^, Jeroen J. G. van Merriënboer^1^, Maryam Asoodar^1^

**Affiliation:** ^1^School of Health Professions Education, Faculty of Health, Medicine & Life sciences, Maastricht University, the Netherlands

^2^Department of Anatomy and Embryology, Maastricht University, Maastricht, The Netherlands

E-mail address of the corresponding author: [h.yu@maastrichtuniversity.nl](mailto:h.yu@maastrichtuniversity.nl)

**Supplementary Figure**

**FIGURE: S1 *Code co-occurrence and coefficient table***


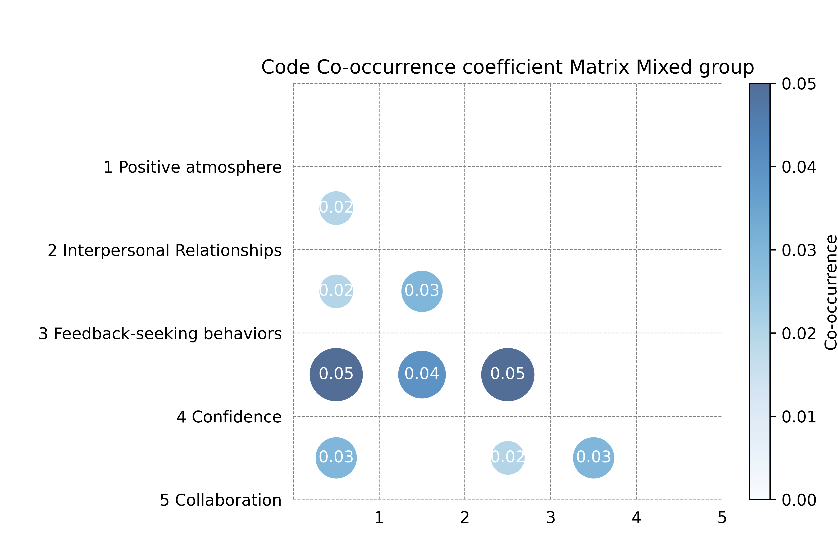

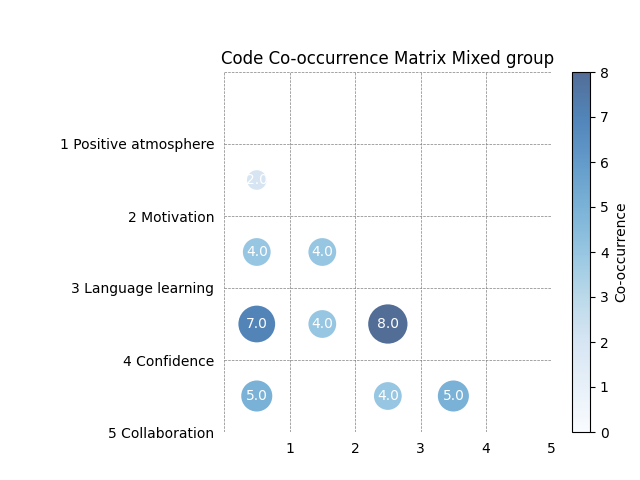

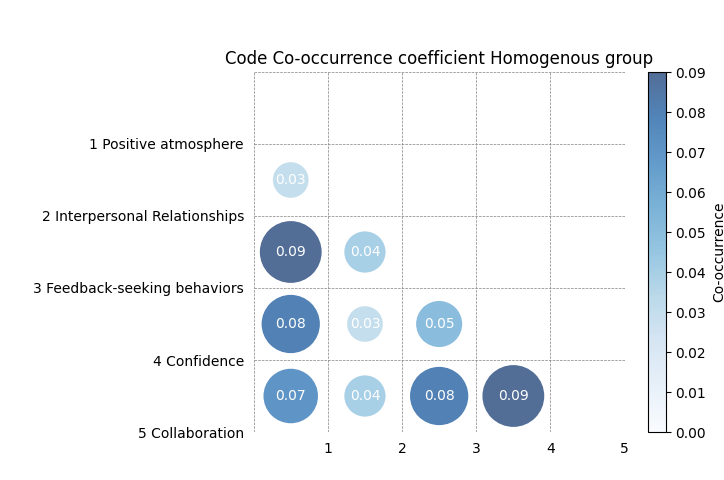

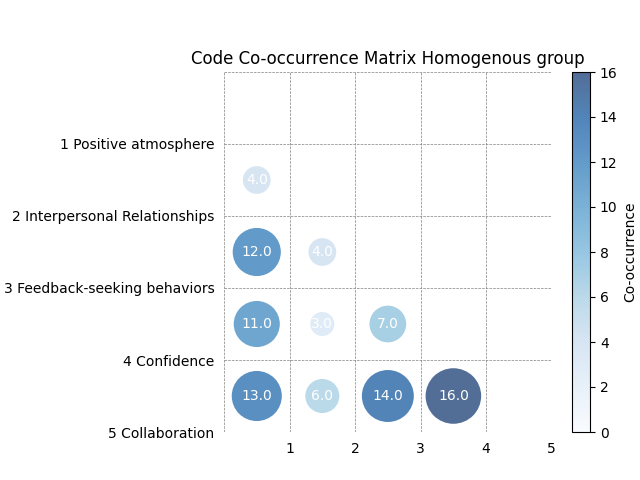

Supplement: Supplementary file 3 — Supplementary file3 (DOCX 621 KB) [file 40670_2024_2138_MOESM3_ESM.docx]
