# Supplementary material for: Impact of Native and Nonnative Study Partners on Medical Students’ Confidence and Collaborative Strategies in Second Language Medical Dutch Learning
Source: Med Sci Educ. 2024 Aug 12;34(6):1445–55. doi: 10.1007/s40670-024-02138-1 (PMC11699018; doi:10.1007/s40670-024-02138-1)
Supplement: Supplementary file 4 — Supplementary file4 (DOCX 31 KB) [file 40670_2024_2138_MOESM4_ESM.docx]

**Supplemental Digital Appendix 4**

**Article Title:** Impact of native and nonnative study partners on medical students’ confidence and collaborative strategies in second language medical Dutch learning

**Journal Name:** Medical Science Educator

**Author Names:** Hao Yu^1*^, S. Eleonore Köhler^2^, Fatemeh Janesarvatan^1^, Jeroen J. G. van Merriënboer^1^, Maryam Asoodar^1^

**Affiliation:** ^1^School of Health Professions Education, Faculty of Health, Medicine & Life sciences, Maastricht University, the Netherlands

^2^Department of Anatomy and Embryology, Maastricht University, Maastricht, The Netherlands

E-mail address of the corresponding author: [h.yu@maastrichtuniversity.nl](mailto:h.yu@maastrichtuniversity.nl)

**Supplementary Table**

**TABLE: S*1 Collaborative strategies***

The content analysis yielding six dimensions of collaborative strategies from each student. The summation of the result presented in figure 4.

| **student 1** | **language and spc** | **communication** | **comparison** | **interaction** | **personal growth** | **drawbacks** |
| --- | --- | --- | --- | --- | --- | --- |
|  | Language learning | Language proficiency: Communication skills | Comparison: Preference | Feedback: Constructive criticism | Rigid thinking | Pressure: Performance pressure |
|  | Knowledge acquisition | Positive feedback | Comparison: Comparing | Psychological factors: Motivation | Perceived usefulness | |
|  | Language proficiency: Language acquisition | Cultural background | Comparison: Feedback | Everyday life: Education | Self-sufficiency |  |
|  | Performance: Expertise |  |  | Sharing knowledge | Confidence building | |
|  | Professionalism |  |  | Social Skills: Respect | Preparation |  |
|  | Monotonous |  |  | Collaboration |  |  |
|  | Patient care |  |  |  |  |  |
|  |  |  |  |  |  |  |
|  | 7 | 3 | 3 | 6 | 5 | 1 |
| **student 2** | **language and spc** | **communication** | **comparison** | **interaction** | **personal growth** | **drawbacks** |
|  | Language learning | Helpfulness | Comparison: Feedback | Feedback: Making Mistakes | Effectiveness |  |
|  | Language proficiency: Foreign Language | Performance: Positivity | | Positive feedback | Self-improvement | |
|  | Language Transition | Multicultural Experience | | Supportiveness | Language proficiency: Personal growth | |
|  | Empathy | Respectful |  |  |  |  |
|  |  | Appreciation |  |  |  |  |
|  |  | Approval |  |  |  |  |
|  | 4 | 6 | 1 | 3 | 3 | 0 |
| **student 3** | **language and spc** | **communication** | **comparison** | **interaction** | **personal growth** | **drawbacks** |
|  | ·Communication: Consultation | ·Fluency | ·Comparison: Feedback | ·Feedback: Positive evaluation | ·Reflection | ·Difficulty with language comprehension |
|  | ·Validation of skills |  | ·Comparison: Comparison | ·Collaboration | ·Improvement |  |
|  | 2 | 0 | 2 | 2 | 2 | 1 |
| **student 4** | **language and spc** | **communication** | **comparison** | **interaction** | **personal growth** | **drawbacks** |
|  | ·Language learning |  |  | ·Communication: Observation | ·Self-reflection | ·Psychological factors: Ambivalence |
|  | ·Performance: Simplification | |  |  | ·Reflection |  |
|  | ·Recalling past events or experiences | |  |  |  |  |
|  | 3 | 0 | 0 | 1 | 2 | 1 |
| **student 5** | **language and spc** | **communication** | **comparison** | **interaction** | **personal growth** | **drawbacks** |
|  | ·Insider knowledge | ·Clarity of communication | ·Comparison: Feedback | ·Supportiveness | ·Goal-setting | ·Ambiguity |
|  | ·Patient-therapist relationship | ·Appreciation | ·Comparison: Comparative analysis | ·Collaboration | ·Recognition: Acknowledgment | ·Performance: Uncertainty |
|  | ·Interpersonal relations | ·Social influence |  | ·Communication: Observation | ·Recognition: Gratitude | ·Difficulty with language comprehension |
|  | ·Performance: Openness | ·Everyday life: Community | | ·Psychological factors: Cultural influence | ·Supportive | ·Inadequacy |
|  |  | ·Preference for in-person communication | |  | ·Analytical thinking | ·Anxiety |
|  |  | ·Social Skills: Cooperation | |  | ·Self-improvement | ·Social isolation/loneliness |
|  |  |  |  |  |  | ·Language proficiency: Language barriers |
|  | 4 | 6 | 2 | 4 | 6 | 7 |
| **student 6** | **language and spc** | **communication** | **comparison** | **interaction** | **personal growth** | **drawbacks** |
|  | ·Language proficiency (2) | ·Communication: Active listening | ·Comparison: Feedback | ·Feedback: Positive evaluation | | ·Judgment |
|  | ·Learning orientation | ·Communication: Observation | | ·Collaboration |  |  |
|  | ·Performance feedback |  |  | ·Group dynamics |  |  |
|  | 3 | 2 | 2 | 3 | 0 | 1 |
| **student 7** | **language and spc** | **communication** | **comparison** | **interaction** | **personal growth** | **drawbacks** |
|  | ·Learning process | ·Communication: Observation | ·Comparison: Feedback | ·Seeking feedback | ·Goal setting | ·Performance: Uncertainty |
|  | ·Skill development |  |  | ·Encouragement | ·Inspiration |  |
|  | ·Language proficiency |  |  | ·Cultural background | ·Everyday life: Education | |
|  | ·Validation |  |  | ·Mutual learning | ·Proud of someone/something | |
|  | ·Language proficiency: Language barriers | |  | ·Shared struggles | ·Recognition: Praise | |
|  | ·Appreciation |  |  | ·Positive feedback | ·Self-reflection |  |
|  | ·Benefit of second opinion | |  |  |  |  |
|  | 7 | 1 | 1 | 6 | 6 | 1 |
| **student 8** | **language and spc** | **communication** | **comparison** | **interaction** | **personal growth** | **drawbacks** |
|  | ·Language proficiency |  |  | ·Comforting | ·Psychological factors: Positive reinforcement | ·Difficulty with language comprehension |
|  | ·Medical knowledge |  | ·Comparison: Feedback | ·Mutual benefit | ·Inadequacy | ·Self-doubt |
|  | ·Language proficiency: Language switching | |  | ·Positive feedback |  | ·Performance: Uncertainty |
|  | ·Validation |  |  | ·Everyday life: Education | |  |
|  | ·Improvement suggestions | |  | ·Encouragement |  |  |
|  | ·Grammar correction |  |  |  |  |  |
|  | ·Focus on strengths |  |  |  |  |  |
|  | ·Agreement/Confirmation | |  |  |  |  |
|  | ·Performance: Evolution/Improvement | |  |  |  |  |
|  |  |  |  |  |  |  |
|  | 9 | 0 | 1 | 5 | 2 | 3 |
| **student 9** | **language and spc** | **communication** | **comparison** | **interaction** | **personal growth** | **drawbacks** |
|  | ·Language proficiency: Language barriers | ·Communication: Clarification needed | ·Comparison: Feedback | ·Advice | ·Confidence building | ·Feedback: Lack of feedback |
|  | ·Performance: Effort | ·Language proficiency: Communication skills | | ·Positive feedback | ·Comforting | ·Ineffectiveness of feedback |
|  | ·Preparation | ·Communication: Communication (2) | | ·Collaboration |  | ·Difficulty with language comprehension |
|  | ·Customization | ·Teamwork |  | ·Offering help |  | ·Psychological factors: Frustration |
|  | ·Specialization |  |  | ·Warning |  | ·Performance: Uncertainty |
|  | ·Patient care |  |  | ·Supportive relationship | |  |
|  | ·Instruction |  |  | ·Encouragement |  |  |
|  | ·Appreciation |  |  | ·Reassurance |  |  |
|  | ·Language learning difficulties | |  |  |  |  |
|  | 9 | 4 | 1 | 8 | 2 | 5 |
| **student 10** | **language and spc** | **communication** | **comparison** | **interaction** | **personal growth** | **drawbacks** |
|  | ·Language proficiency: Proficiency in language | ·Clarity of communication | ·Comparison: Comparing oneself to others | | ·Recognition: Achievement | ·Performance: Uncertainty |
|  | ·Language learning | ·Performance: Effective communication | |  | ·Ability to improvise | ·Language proficiency: Language difficulties |
|  | ·Necessity of learning phrases/specifities of foreign languages | ·Cultural background |  |  |  | ·Communication difficulties |
|  | ·Productivity | ·Social Skills: Friendship | |  |  | ·Confusion |
|  | ·Linguistic abilities |  |  |  |  | ·Overwhelmed |
|  | ·Appreciation |  |  |  |  |  |
|  | ·Performance: Accountability | |  |  |  |  |
|  | 7 | 4 | 1 | 0 | 2 | 5 |
| **student 11** | **language and spc** | **communication** | **comparison** | **interaction** | **personal growth** | **drawbacks** |
|  | ·Observation Skills | ·Social relationships | ·Comparison: Feedback | ·Constructive Feedback | ·Encouragement | ·Language barrier |
|  | ·Appreciation | ·Cultural background | ·Communication: Observation | ·Giving feedback | ·Self-improvement | ·Personal opinion |
|  | ·Approval | ·Psychological factors: Positive reinforcement | | ·Feedback: Constructive criticism | |  |
|  | ·Evaluation | ·Positive feedback |  |  |  |  |
|  | 4 | 4 | 3 | 3 | 2 | 2 |
| **student 12** | **language and spc** | **communication** | **comparison** | **interaction** | **personal growth** | **drawbacks** |
|  | ·Language proficiency: Learning process | ·Communication: Communication (2) | ·Comparison: Structure | ·Feedback: Positive evaluation | ·Trust | ·Limitations |
|  | ·Error correction | ·Performance: Clear communication | ·Comparison: Feedback | ·Feedback: Constructive criticism | ·Satisfaction | ·Feedback: Negative perception |
|  | ·Rapport building | ·Positive feedback | ·Comparison: Preference | ·Teamwork | ·Positive learning experience | |
|  | ·Performance: Competence | ·Performance: Positivity | |  | ·Everyday life: Routine | |
|  | ·Empathy | ·Social Skills: Helping others | |  | ·Psychological factors: Autonomy | |
|  | ·Appreciation | ·Language proficiency: Cultural knowledge | |  | ·Improvement |  |
|  | ·Teacher-student relationship | |  |  | ·Analytical thinking | |
|  | ·Performance: Efficiency |  |  |  | ·Desire to improve | |
|  | ·Effectiveness |  |  |  | ·Rules and expectations | |
|  | 9 | 6 | 3 | 3 | 9 | 2 |
